# Disability pension receipt in young adults: an analysis of the Swiss Social protection and labour market (SESAM) data

**DOI:** 10.1186/s12889-019-7098-1

**Published:** 2019-06-26

**Authors:** Szilvia Altwicker-Hámori, Julia Dratva

**Affiliations:** 1 Research Unit for Health Sciences, ZHAW School of Health Professions, Technikumstrasse 81, 8400 Winterthur, Switzerland; 2 Faculty of Medicine, University of Basel, Klingelbergstrasse 61, 4056 Basel, Switzerland

**Keywords:** Disability pension, Young adults, Switzerland, Logistic regression

## Abstract

**Background:**

There has been an overall decreasing trend in the inflow into disability pension in Switzerland since 2003 with the exception of young adults. Disablement in young adulthood reflects a particularly critical phenomenon given the potentially far-reaching long-term social, economic and health consequences. The aim of this study was therefore to identify factors for disability pension in young adults aged 18–39, living in Switzerland.

**Methods:**

We used the 2010–2015 cross-sections of the Social protection and labour market; a unique dataset linking microdata from the Swiss Labour Force Survey, the Swiss Central Compensation Office Register, and the Unemployment Insurance Register. Multiple logistic regression was employed to explore the association between demographic, socioeconomic, and health factors and disability pension in young adults living in Switzerland with long-term activity limitation (*N* = 5306). Alternative specifications of the benchmark model were estimated as robustness checks; and subsample analyses were conducted excluding (i) those aged 18–24 and (ii) those with partial disability pension.

**Results:**

Our regression results showed that those living without a working partner (OR 2.11; 95% CI 1.51–2.94) and without a child aged 0–14 (OR 2.15; 95% CI 1.48–3.12), born in Switzerland (OR 2.68; 95% CI 1.87–3.84), of higher age (OR 1.16; 95% CI 1.12–1.19), having completed at most lower secondary school (OR 3.26; 95% CI 2.24–4.76), lacking income throughout the four-year period prior to interview (OR 3.94; 95% CI 2.70–5.75), suffering from chronic illness (OR 4.52; 95% CI 2.83–7.19), and severe long-term activity limitation (OR 4.52; 95% CI 2.83–7.19) had higher odds of DP. Our findings were robust to alternative specifications and subsamples; and the alternative specifications revealed differences by learnt occupation, with highest odds for those without an occupational qualification (OR 5.93; 95% CI 3.72–9.46; *p*-value 0.000) and for those in ‘Manufacturing’ (OR 3.59; 95% CI 1.91–6.71) relative to ‘Health, education, culture, and science’.

**Conclusions:**

Most importantly, our results showed that educational and employment factors are of high relevance, as well as chronic morbidity and severe long-term activity limitation. From a policy perspective, early intervention should thus focus on the attainment of vocational and academic qualifications beyond the lower secondary level, facilitating school-to-work transition and labour market integration.

## Background

There has been an overall decreasing trend in the inflow into disability pension (DP) in Switzerland since 2003 with the exception of young adults, who experienced no such decrease [1]. Disablement in young adulthood reflects a particularly critical phenomenon. The vast majority of individuals do not exit DP [2, 3]; and if they do leave  DP, they are likely to move onto another benefit [3]. Subsequently, they are unlikely to reap the benefits of paid work such as autonomy, social contacts with colleagues, and social support [4]. Moreover, DP may have detrimental effects on the individual’s health [5–9] and health behavior [10]. For instance, Swedish studies showed that DP granted in young adulthood due to common mental disorders are associated with subsequent suicidal behavior [8, 9]; and called particular attention to younger individuals (aged 18–24 years) on DP due to anxiety disorders because of their higher suicide risk [8]. The labour market integration of individuals with disabilities, especially at young ages, is also essential from a societal perspective in light of labour market shortages [11] and population aging [3].

Enhancing the employment opportunities of individuals with disabilities has thus been on the political agenda in Switzerland for over a decade. In particular, there have been three revisions to the Federal Act on Disability Insurance between 2004 and 2012 aimed at the labour market (re-)integration of disabled individuals and the consequent increase in their autonomy, thereby reducing the inflow into DP [12]. In 2004 the 4th revision introduced the assistance compensation in order to increase the autonomy of individuals with disabilities [13]; in 2008 the 5th revision developed measures for early detection, early intervention, and integration in order to identify affected persons as early as possible and to support them in keeping their current jobs [14]. The 6(a)^th^ revision, which came into force in 2012, was targeted especially at the labour market reintegration DP recipients [15]. For example, measures were designed to support the reintegration process for 3 years after taking up employment for both employers and disabled individuals [15].

Given the high inflow into DP, coupled with the adverse economic, social and health consequences of disablement, identifying factors for receiving DP in young adulthood appears essential; and understanding the factors for DPs in young adulthood may in turn be valuable when developing preventive and supportive interventions. The factors identified for the general population in the large and growing body of international literature provide a suitable starting point for our analysis. In addition to medical factors, numerous non-medical factors have been shown to be associated with DP, including demographic factors (such as age and country of birth) and socioeconomic factors (for example, educational attainment, income, unemployment, and occupation) [10, 16–20]. Examining which factors apply to young adults living in Switzerland is of great importance; in particular given the evidence that factors vary by age cohort and by institutional setting [10, 21]. In addition, risk factors vary by broad diagnosis groups [10, 18, 22]. Congenital and mental illness diagnoses predominate in DP in younger ages as opposed to musculoskeletal diagnoses in higher ages in Switzerland [23], underscoring the need for a separate analysis in young adults.

Therefore, the aim of the present study was to explore the association between demographic, socioeconomic, and health factors and DP in young adults suffering from long-term activity limitation and living in Switzerland. To the best of our knowledge, we were the first to use the Social protection and labour market (SESAM) – linking microdata from the Swiss Labour Force Survey (SLFS) and different social insurance registers – to analyse this topic, thereby demonstrating the potential of this dataset for the questions at hand. The results contribute to the understanding of the continuously high DP receipts in young adults, a special at-risk group, despite reforms in Switzerland.

## Methods

### Data

For the statistical analysis data was drawn from the SESAM provided by the Swiss Federal Statistical Office (FSO) [24, 25]. The SESAM is a links microdata from the SLFS [26, 27] and different social insurance registers. The SLFS is a telephone household survey, carried out since 1991, providing a wide range of information on labour market situation, educational background, household composition, and demographic characteristics as well as health status. The SLFS adheres to international concepts and definitions, in particular to those employed in the European Union Labour Force Survey [28], thereby enabling comparisons with OECD an EU data. The social insurance registers in SESAM include the following registers: old age and survivors’ insurance; disability pensions; complementary benefits; individual accounts; and unemployment insurance. Linkage is based on the SLFS sample via the respondents’ social insurance number. The SESAM therefore provides a unique opportunity for research in employment, health, and social security. A further advantage of SESAM lies in its sample size. It covers almost 1% of Switzerland’s permanent resident population aged 15 and over; corresponding to Swiss citizens whose main residence is in Switzerland and foreign citizens residing in Switzerland for at least 12 months [26]. In our dataset, around 2% of young adults received a DP; a figure in line with annual Swiss Federal Social Insurance Office (FSIO) statistics [23, 29].

For the current analysis, the following SESAM sources were used: (1) SLFS, (2) individual accounts, and (3) disability pensions. All independent variables used in the statistical analysis, with the exception of annual income and DP, were retrieved from the SLFS source of SESAM. Variables concerning annual income and DP, came from the individual accounts and the disability pensions registers, respectively.

### Sample selection

We defined our sample of interest to include ‘new-DP recipients’ and ‘non-new-DP recipients’ who were (1) aged 18 to 39 years, (2) reported long-term activity limitation, and (3) were not in education or training at survey participation. The age range of 18 to 39 was chosen based on Erikson’s Stages of Psychosocial Development [30]. The lower age limit also represents the minimum age for DP entitlements in Switzerland, thereby providing a suitable cut-off. In line with the FSIO’s definition of new-DP recipients, we considered the first event of a DP within a two-year period [2]. Accordingly, inclusion criteria for the new-DP recipients were that they (1) received a DP in the survey participation year and (2) were not DP recipients 12 months preceding their survey participation. Inclusion criteria for non-new-DP recipients were that they (1) did not receive a DP in the survey participation year; (2) were in paid employment in the survey participation year; and (3) were not DP recipients 12 months preceding their survey participation. The group of non-new-DP recipients was restricted to individuals in paid employment to ensure homogeneity in terms of employment status; that is, they were all successfully integrated in the labour market without relying on DP. For simplicity, in the remainder of this paper we refer to the two groups as ‘DP recipients’ and ‘non-DP recipients’, respectively.

**Fig. 1:**
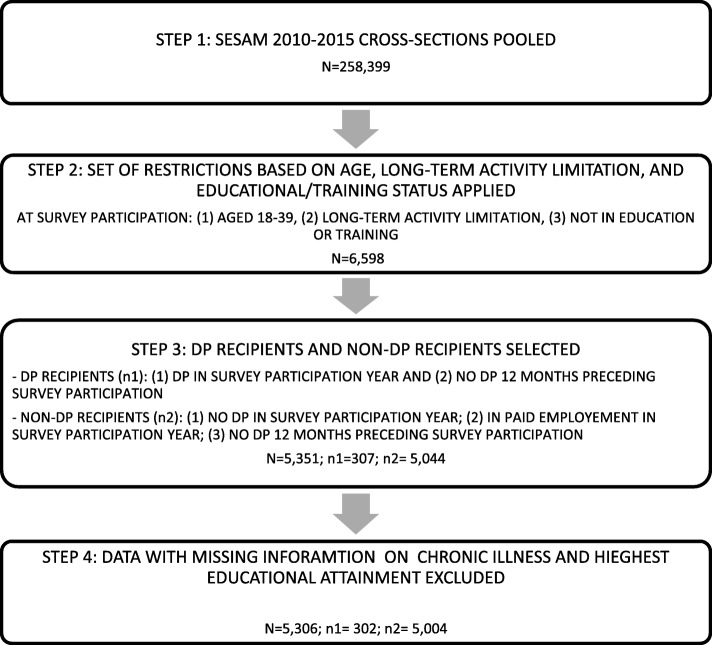
Sample selection procedure. Legends: Source: SESAM, FSO

To identify our sample of interest in SESAM, we carried out four steps summarized in Fig. 1. We first generated an independently pooled cross-sectional dataset covering the period of 2010 to 2015 (*N* = 258,399). We restricted the analysis to these 6 years because of the availability of the Minimum European Health Module (MEHM) in SESAM. The MEHM consists of three global questions concerning three different health domains, namely, (1) self-perceived health, (2) chronic morbidity, and (3) long-term activity limitation [31]. The third domain was essential for the identification of our sample of interest. In particular, we selected individuals on the basis of the answer to the following question: ‘For at least the past six months, to what extent have you been limited because of a health problem in activities people usually do?’ [32]. Individuals answering ‘severely limited’ or ‘limited but not severely’ were eligible. Furthermore, we selected individuals fulfilling the inclusion criterion described above regarding age and the exclusion criterion regarding educational/training status (*N* = 6598). We then applied the next set of restrictions concerning employment and DP status described above to arrive at the sample of DP recipients and non-DP recipients (*N* = 5351). Finally, respondents with missing information on key independent variables, corresponding to the variables in the benchmark model (see Section ‘Independent Variables’), were dropped from the sample; amounting to less than 1% of the sample (*N* = 45). Specifically, 32 observations were omitted due to missing data on chronic illness; and 13 observations were omitted due to missing data on the level of highest educational attainment. Our final sample contained 5306 individuals.

### Outcome variable

Our outcome variable was the first event of a DP between 2010 and 2015. DPs in Switzerland are awarded to claimants once rehabilitation measures aimed at restoring, maintaining or improving their earning capacity or their day-to-day activities have been exhausted [33]. DPs can be full or partial depending on the degree of disability. Claimants with at least 70%, 60–69%, 50–59%, and 40–49% disability are eligible for a full, three-quarter, half, and quarter DP, respectively [33]. In our benchmark analysis we considered all types of DPs as DP.

### Independent variables

Our benchmark model included information on sex, country of birth, household structure, age, education, income, and health. Dichotomous variables indicating the respondent’s sex (‘Male’ versus ‘Female’); country of birth (‘Switzerland’ versus ‘Outside of Switzerland’); the presence or absence of own children or step-children, aged 14 years or younger, living in the same household were included. A dichotomous variable indicating the presence or absence of an employed partner (married or cohabiting) in the household was generated based on (1) information on the relationship of household members to the reference person and (2) the respective household member’s employment status. The employed category combined the following: employees, self-employed, apprentices, and family members working in family business. Age was used as a continuous variable.

The 12 categories for highest educational attainment, classified based on the Swiss System for Household and Personal Statistics (SHAPE) [34], were dichotomised into ‘Lower secondary’ as opposed to ‘Upper secondary and tertiary’. Given that the completion of lower secondary education in Switzerland marks the end of compulsory education, it provides a suitable cut-off.

The SESAM contains information on the amount of total annual gross income at the individual level; whereby total income includes earnings as well as compensation for the loss of earnings due to disability, unemployment, maternity leave, and military service. The amount of total annual gross income was available for 4 years prior to the survey year minus 1 year. For example, for 2015 survey participants their total annual gross income was available for 2010, 2011, 2012, and 2013. We combined this yearly income information to generate a dichotomous variable distinguishing between respondents who have received income at least once within the four-year-period and those who have not. For example, for 2015 respondents, the latter variable indicated whether they received income at least once between 2010 and 2013 or not. For simplicity, we refer to the four-year period prior to survey year minus 1 year as the four-year period prior to interview in the remainder of this  paper.

The second and third MEHM domains were used to capture the respondents’ health. An indicator variable for the presence or absence of chronic conditions (Domain 2) was generated based on the question ‘Do you have any longstanding illness or health problem?’, whereby longstanding was defined as lasting for at least 6 months prior to the survey or likely to affect the respondent for at least 6 months after the survey [32]. Second, a dichotomous variable indicating the severity of the respondent’s long-term activity limitation was generated based on the third MEHM domain differentiating between ‘Severely limited’ versus ‘Limited but not severely’.

Finally, we created a time period dummy variable (‘2010–2011’ versus ‘2012–2015’) in order to capture the coming into force of the 6(a)^th^ revision to the Federal Act on Disability Insurance in 2012.

In an additional model (described in detail in Section ‘Analysis’), we replaced highest educational attainment by learnt occupation, categorised based on the Swiss Standard Classification of Occupations (SSCO) 2000 [35]. The SSCO 2000 classifies 20,000 occupations using five-digit codes according to economic activity. The five-digit occupations can be aggregated at the highest level into ‘Divisions of professions’ (one-digit level). Accordingly, we aggregated the five-digit codes into ten categories as follows: (1) ‘Not applicable’; (2) ‘Agriculture, forestry, and livestock production’ [henceforth ‘Agriculture’]; (3) ‘Manufacturing’; (4) ‘Technical activities and ICT’, (5) ‘Construction and mining’; (6) ‘Trade and transport’; (7) ‘Hotels and catering, and other personal services [henceforth ‘Personal services’]; (8) ‘Management, administration, finance, insurance, and law’ [henceforth ‘Business and administration’]; (9) ‘Health, education, culture, and science’; and (10) ‘Not classifiable’. Given that the learnt occupational categories used in this study are based on the Swiss rather than on an international classification system of occupations, numerous points merit comment. The first one concerns the composition of the ‘Not applicable’ category. In the full 18–39-year-old sample, the ‘Not applicable’ category almost exclusively included individuals who did not earn an occupational degree. More specifically, individuals who at most completed lower secondary education (57%), general upper secondary education (24%), and short courses (less than 2 years in duration) at the upper secondary level or other programs not leading to a specific occupational degree (16%); have not completed compulsory school (3%); or for whom learnt occupational information was missing (less than 1%) belonged to the ‘Not applicable’ category. The proportion of general upper secondary graduates was higher in non-DP recipients (26%) than in DP recipients (7%); whereas the proportion of those who have left school without a degree was lower (2 and 7%, respectively). The second aspect concerns the composition of the ‘Not classifiable’ category. The ‘Not classifiable’ category included mostly non-classifiable occupations at the upper secondary level (83%), followed by those at the tertiary level (16%); non-classifiable manual occupations and those with an unknown degree level amounted to merely 1%. Third, the ‘Health, education, culture, and science’ category was not as heterogeneous as potentially suggested by its title: it was mainly composed of health care professionals (33%; mostly nurses), teaching professionals (29%; mostly daycare, primary and lower secondary school teachers), and social scientists (28%; mostly economists); and merely 10% were cultural professionals (such as musicians or graphic designers). Non-DP recipients and DP recipients differed substantially only in the share of health care professionals (33 and 56%, respectively) and scientists (29 and 19%, respectively). Finally, in terms of educational attainment ‘Technical activities and ICT’ and ‘Health, education, culture, and science’ ranked highest (72% tertiary graduates in each category), followed by ‘Business and administration’ (38% tertiary graduates); and in all categories, the share of tertiary graduates was lower among DP recipients than among non-DP recipients.

In our final model (described in detail in Section ‘Analysis’), a dichotomous variable capturing marital status (‘Single’ versus ‘Married or registered partnership’) was included as an alternative to the variable indicating a working partner’s presence or absence in the household; whereby the single category combined (1) single, (2) divorced, (3) legally separated, (4) widowed, and (5) dissolved partnership.

### Analysis

Characteristics of DP recipients and non-DP recipients were compared using Pearson’s chi-square tests or Wilcoxon-Mann-Whitney test, as appropriate. Multiple logistic regression models were applied to examine the associations between demographic, socioeconomic, and health characteristics and DP. Model 1 represented our benchmark model; and included variables capturing the individual’s sex, country of birth, working partner’s presence in the household, presence of a child aged 14 years or younger in the household, age, highest educational attainment, income, the presence of chronic illness, severity of the long-term activity limitation, and time period. Models 2 and 3 represented alternative specifications of Model 1: in Model 2 highest educational attainment was replaced by a set of indicator variables for learnt occupation and in Model 3 the indicator variable for the working partner’s presence in the household was replaced by an indicator variable for marital status.

Subsample analysis was carried out based on age. We carried out the estimation in the subsample of 25-to-39-year-olds, representing the lower segment of the prime-age working population. Raising the lower age bound to age 25 mitigates the possibility that the respondents may still go onto tertiary education, thereby generating a more homogeneous sample in terms of employment possibilities as well as educational career. Moreover, we carried out the analysis using full DP as our outcome in order to focus on individuals with the highest degree of incapacity to work or carry out day-to-day activities.

In further models not reported in the present study, we added variables indicating homeownership as a proxy for economic status and regions of residence at the NUTS-2 level (Lake Geneva Region, Espace Mittelland, Northwestern Switzerland, Zurich, Eastern Switzerland, Central Switzerland and Ticino) to our benchmark model (Model 1), respectively. The estimated coefficients on the additional control variables were not statistically significant and their inclusion did not affect the remaining coefficient estimates. The only exception was the estimated coefficient on Eastern Switzerland; those residing in Eastern Switzerland had higher odds of DP receipt (OR 1.74; 95% CI 1.11–2.73; *p*-value 0.016) than those residing in Espace Mittelland. Moreover, in light of the varying upper age limit for young adults [36, 37], we carried out the regression analysis in the subsample aged 18–35 (*N* = 3813); the estimation results were in line with those aged 18–39.

Individual weights, provided in the SESAM, were used in all statistical analyses. Variance inflator factor was used to assess multicollinearity in all estimated models; there was no indication of multicollinearity. Results of the multiple logistic regression models are presented as odds ratios (OR) with *p*-values and 95% confidence intervals (95% CI). A p-value of ≤5% was regarded as statistically significant in all analyses. All figures are reported in line with FSO regulations. Accordingly, (1) statistics based on less than five observations are not reported and (2) statistics based on more than four but less than 50 observations are reported in brackets; in percentage calculations, the regulation applies to the numerator and not the denominator. All statistical analyses were conducted using Stata 14.

## Results

### Descriptive statistics

Characteristics of respondents aged 18–39 by DP are presented in Table 1. The proportion of females and males in the full sample was approximately equal (55% females; 45% males). The majority of the full sample did not have a working partner (59%) or a child aged 0–14 years (72%) living in the same household, was born in Switzerland (74%), had an upper secondary or tertiary degree (84%), had received income at least in 1 year within the four-year period prior to interview (72%), reported chronic illness (66%) and long-term activity limitation (89%), was drawn from the 2012–2015 cross-sections (84%), and was single (69%). Mean age was around 29 years. For slightly more than a quarter (28%) of the full sample learnt occupation was recorded as ‘Not applicable’ either because the respondent had not finished school or had earned a general educational degree so that a learnt occupation could not be assigned. Around 20% had learnt an occupation within the field of ‘Health, education, culture, and science’, around 10% in ‘Business and administration’, around 10% in ‘Trade and transport’, around 8% in ‘Manufacturing’, around 7% in ‘Technical activities and ICT’, around 5% in ‘Construction and mining’, around 5% in ‘Personal services’, and around 2% in ‘Agriculture’. For approximately 4% of the full sample the learnt occupation could not be classified.

**Table 1:** Descriptive statistics for respondents aged 18–39 by DP (weighted %)

	All (*N* = 5306)	Non-DP recipients (*n* = 5004)	DP recipients (*n* = 302)	*P*-value*
Sex				
Female	54.89	55.08	51.66	0.316
Male	45.11	44.92	48.34	
Working partner in household				
Yes	41.17	42.00	26.93	< 0.001
No	58.83	58.00	73.07	
Child aged 0–14 years in household				
Yes	28.45	28.85	21.71	0.011
No	71.55	71.15	78.29	
Country of birth				
Outside of Switzerland	25.90	26.38	17.61	0.001
Switzerland	74.10	73.62	82.39	
Mean age [standard deviation]				
	29.48 [6.27]	29.38 [6.28]	31.16 [5.81]	< 0.001**
Highest degree attained				
Upper secondary or tertiary	83.55	84.55	66.42	< 0.001
Lower secondary	16.45	15.45	33.58	
Income during 4 years prior to interview				
Yes	71.87	73.10	50.92	< 0.001
No	28.13	26.90	49.08	
Chronic illness				
No	34.29	35.80	(8.59)	< 0.001
Yes	65.71	65.20	91.41	
Long-term activity limitation				
Limited but not severely	89.02	90.47	64.39	< 0.001
Severely limited	10.97	9.53	35.61	
Time period				
2012–2015	83.54	84.60	65.34	< 0.001
2010–2011	16.46	15.40	34.66	
Learnt occupation				
Not applicable	27.63	26.18	52.36	< 0.001
Agriculture	2.03	2.02	(2.26)	
Manufacturing	8.16	8.04	(10.13)	
Technical activities and ICT	7.49	7.81	(2.15)	
Construction and mining	5.04	5.21	(2.17)	
Trade and transport	9.74	9.87	(7.60)	
Personal services	5.08	5.16	(3.85)	
Business and administration	10.32	10.34	(9.88)	
Health, education, culture, and science	20.38	21.14	(7.40)	
Not classifiable	4.12	4.23	(2.21)	
Marital status				
Married or in registered partnership	30.89	31.38	22.54	0.002
Single	69.11	68.62	77.46	

Source: SESAM, FSO. *P-values based on Pearson’s chi-square tests. **z-value based on Wilcoxon-Mann-Whitney test. DP recipients received a DP in the survey participation year and were not DP recipients 12 months preceding their survey participation. Non-DP recipients did not receive a DP in the survey participation year; were in paid employed in the survey participation year; and were not DP recipients 12 months preceding their survey participation. Figures in brackets: Extrapolation based on less than 50 observations. The results should be interpreted with great caution

There were statistically significant differences between non-DP recipients and DP recipients with regard to all the characteristics other than sex. In terms of household structure, a higher proportion of non-DP recipients was living with a working partner (42%) and with a child aged 0–14 years (29%) than DP recipients (27 and 22%, respectively). There were fewer Swiss-born among the non-DP recipients (74%) than among the DP recipients (82%). Non-DP recipients were on average younger (29 versus 31 years) and were better educated: 85% of non-DP recipients earned at least an upper secondary degree; this figure was merely 66% among DP recipients. The proportion of individuals with at least one recorded income within 4 years preceding the interview amounted to 73% in non-DP recipients and only to 51% in DP recipients. Non-DP recipients reported chronic illness and severe long-term activity limitation less often (65 and 10%, respectively) than DP recipients (91 and 36%, respectively). In terms of the learnt-occupational composition, compared to DP recipients, non-DP recipients had a lower proportion in the ‘Not applicable’ category (26% versus 52%). A higher share of non-DP recipients were married or in a registered partnership than DP recipients (31 and 23%, respectively).

Descriptive statistics for the subsample of 25–39-year-olds by DP are reported in Table 2. The differences between non-DP recipients and DP recipients showed the same pattern as in the 18–39-year-old group and were significant, with the exception of sex (*p*-value 0.935) and age (borderline significance; p-value 0.068).

**Table 2:** Descriptive statistics for respondents aged 25–39 by DP (weighted %)

	All (*N* = 4170)	Non-DP recipients (*n* = 3906)	DP recipients (*n* = 264)	*P*-value*
Sex				
Female	54.58	54.60	54.31	0.935
Male	45.42	45.40	45.69	
Working partner in household				
Yes	51.73	53.03	32.06	< 0.001
No	48.27	46.97	67.94	
Child aged 0–14 years in household				
Yes	37.59	38.37	25.80	< 0.001
No	62.41	61.63	74.20	
Country of birth				
Outside of Switzerland	28.61	29.34	17.62	< 0.001
Switzerland	71.39	70.66	82.38	
Mean age [standard deviation]				
	32.41 [4.28]	32.37 [4.28]	33.06 [4.24]	0.068**
Highest degree attained				
Upper secondary or tertiary	89.48	90.57	73.07	< 0.001
Lower secondary	10.52	9.43	26.93	
Income during 4 years prior to interview				
Yes	83.06	85.02	53.50	< 0.001
No	16.94	14.98	46.50	
Chronic illness				
No	33.43	35.07	(8.63)	< 0.001
Yes	66.57	64.93	91.37	
Long-term activity limitation				
Limited but not severely	88.56	89.96	67.46	< 0.001
Severely limited	11.44	10.04	32.54	
Time period				
2012–2015	82.99	84.14	65.65	< 0.001
2010–2011	17.01	15.86	34.35	
Learnt occupation				
Not applicable	20.01	18.28	46.23	< 0.001
Agriculture	2.31	2.32	(2.07)	
Manufacturing	8.73	8.64	(10.18)	
Technical activities and ICT	9.36	9.81	(2.57)	
Construction and mining	4.65	4.78	(2.60)	
Trade and transport	10.22	10.34	(8.52)	
Personal services	5.03	5.06	(4.61)	
Business and administration	11.66	11.65	(11.86)	
Health, education, culture, and science	24.47	25.5	(8.88)	
Not classifiable	3.56	3.63	(2.47)	
Marital status				
Married or in registered partnership	40.14	41.02	26.79	0.002
Single	59.86	58.98	73.21	

Source: SESAM, FSO. *P-values based on Pearson’s chi-square tests. **z-value based on Wilcoxon-Mann-Whitney test. DP recipients received a DP in the survey participation year and were not DP recipients 12 months preceding their survey participation. Non-DP recipients did not receive a DP in the survey participation year; were in paid employed in the survey participation year; and were not DP recipients 12 months preceding their survey participation. Figures in brackets: Extrapolation based on less than 50 observations. The results should be interpreted with great caution

Descriptive statistics for the subsample in which the outcome was restricted to full DP are reported in Table 3. The differences between non-DP recipients and full-DP recipients were in line with those between non-DP recipients and DP-recipients; and these differences were significant with the exception of sex (borderline significance; *p*-value 0.075).

**Table 3:** Descriptive statistics for respondents aged 18–39 by full DP (weighted %)

	All (*N* = 5196)	Non-DP recipients (*n* = 5004)	Full-DP recipients (*n* = 192)	*P*-value*
Sex				
Female	54.80	55.08	47.60	0.075
Male	45.20	44.92	52.40	
Working partner in household				
Yes	41.11	42.00	(17.85)	< 0.001
No	58.89	58.00	82.15	
Child aged 0–14 years in household				
Yes	28.43	28.85	(15.17)	< 0.001
No	71.66	71.15	84.83	
Country of birth				
Outside of Switzerland	26.06	26.38	(17.02)	0.004
Switzerland	73.96	73.62	82.98	
Mean age [standard deviation]				
	29.41 [6.27]	29.38 [6.28]	30.00 [5.98]	< 0.001**
Highest degree attained				
Upper secondary or tertiary	83.65	84.55	60.00	< 0.001
Lower secondary	16.35	15.45	40.00	
Income during 4 years prior to interview				
Yes	72.05	73.10	44.65	< 0.001
No	27.95	26.90	55.35	
Chronic illness				
No	34.85	35.80	(10.18)	< 0.001
Yes	65.15	65.20	89.82	
Long-term activity limitation				
Limited but not severely	89.38	90.47	61.11	< 0.001
Severely limited	10.62	9.53	38.89	
Time period				
2012–2015	83.76	84.60	61.89	< 0.001
2010–2011	16.24	15.40	38.11	
Learnt occupation				
Not applicable	27.63	26.18	61.38	< 0.001
Agriculture	2.03	2.02	(2.11)	
Manufacturing	8.16	8.04	(7.41)	
Technical activities and ICT	7.49	7.81	X	
Construction and mining	5.04	5.21	X	
Trade and transport	9.74	9.87	(5.16)	
Personal services	5.08	5.16	(3.85)	
Business and administration	10.32	10.34	(9.56)	
Health, education, culture, and science	20.38	21.14	(5.18)	
Not classifiable	4.12	4.23	X	
Marital status				
Married or in registered partnership	30.78	31.38	(15.28)	0.002
Single	69.22	68.62	84.72	

Source: SESAM, FSO. *P-values based on Pearson’s chi-square tests. **z-value based on Wilcoxon-Mann-Whitney test. Full-DP recipients  received a full DP in the survey participation year and were not DP recipients 12 months preceding their survey participation. Non-DP recipients did not receive a DP in the survey participation year; were in paid employed in the survey participation year; and were not DP recipients 12 months preceding their survey participation. Figures in brackets: Extrapolation based on less than 50 observations. The results should be interpreted with great caution. X: Extrapolation based on less than five observations. The results cannot be published for data protection reasons

The SESAM contains information on the main cause of DP, presented in Table 4 by age group and DP type. The sample statistics confirm the predominance of mental and congenital diseases in DP granted in younger ages: in 18–39-year-old DP recipients, the leading cause of DP were mental disorders (51%), followed by congenital disorders (25%). Musculoskeletal disorders/injuries accounted for merely 9% of main DP cause in this group. The ranking remained identical in our subsamples of 25–39-year-old DP recipients and 18–39-year-old full-DP recipients. The fraction of individuals with congenital disorders was highest in full-DP recipients (31%); thereby augmenting FSIO statistics for individuals aged 18–64 according to which congenital disorders as main DP cause were more common in full-DP recipients than in DP recipients (covering all DP types) under the period under analysis [2, 38, 39].

**Table 4:** Distribution of main DP cause by age group and DP type (weighted %)

Main cause of DP	DP recipients aged 18–39 (*N* = 302)	DP recipients aged 25–39 (*n* = 264)	Full-DP-recipients aged 18–39 (*n* = 192)
Disorder			
Congenital	25.23	20.82	31.34
Mental	51.15	53.56	54.46
Nervous system	(6.74)	(6.90)	(4.79)
Musculoskeletal	(7.08)	(7.55)	(4.29)
Other	(6.00)	(6.62)	(3.58)
Accident			
Musculoskeletal	(2.08)	(2.49)	X
Other	(1.72)	(2.07)	X

Source: SESAM, FSO. DP recipients received a DP in the survey participation year and were not DP recipients 12 months preceding their survey participation. Full-DP recipients received a full DP in the survey participation year and were not DP recipients 12 months preceding their survey participation. Figures in brackets: Extrapolation based on less than 50 observations. The results should be interpreted with great caution. X: Extrapolation based on less than five observations. The results cannot be published for data protection reasons

### Regression analysis

Table 5 presents the results of the regression analysis for the sample of 18–39-year-olds. Estimates of our benchmark model (Model 1) indicated that individuals without a working partner in the same household had higher odds of DP (OR 2.11; 95% CI 1.51–2.94; p-value 0.000) than those living with a working partner. Individuals without a child aged 0–14 years living in the same household were also more likely (OR 2.15; 95% CI 1.48–3.12; *p*-value 0.000) to receive a DP than their counterparts living with a child aged 0–14 years. Individuals born in Switzerland were more likely to receive a DP than those born outside of Switzerland (OR 2.68; 95% CI 1.87–3.84; *p*-value 0.000). Age was associated with slightly higher odds of DP (OR 1.16; 95% CI 1.12–1.19; p-value 0.000). Respondents completing at most lower secondary school had higher odds of a DP compared to those with at least an upper secondary school degree (OR 3.26; 95% CI 2.24–4.76; *p*-value 0.000). Individuals without income during four years prior to the interview had higher odds of a DP compared to their counterparts who had at least one recorded income within this four-year period (OR 3.94; 95% CI 2.70–5.75; *p*-value 0.000). Individuals suffering from chronic illness and severe long-term activity limitation were more likely (OR 4.52; 95% CI 2.83–7.19; *p*-value 0.000 and OR 4.24; 95% CI 3.10–5.81; p-value 0.000, respectively) to receive a DP than their counterparts not suffering from a chronic illness and with merely limited long-term activity limitation. 2010–2011 survey respondents were more likely (OR 1.42; 95% CI 1.05–1.92; *p*-value 0.022) to be in receipt of a DP than 2012–2015 survey respondents. No statistically significant association was found between sex and DP. Turning to Model 2, the estimates for the learnt occupation dummies showed that relative to individuals in the ‘Health, education, culture, and science’ category, those in the ‘Not applicable’ (OR 5.93; 95% CI 3.72–9.46; *p*-value 0.000), ‘Manufacturing’ (OR 3.59; 95% CI 1.91–6.71; p-value 0.000), ‘Trade and transport’ (OR 2.14; 95% CI 1.16–3.96; *p*-value 0.015), ‘Personal services’ (OR 2.26; 95% CI 1.00–5.09; *p*-value 0.050), and ‘Business and administration’ (OR 2.44; 95% CI 1.40–4.23; *p*-value 0.002) categories had higher odds of DP. The coefficient estimate for ‘Agriculture’ was borderline significant (OR 2.61; 95% CI 0.95–7.17; *p*-value 0.063). The remaining coefficient estimates stayed robust in Model 2, with the exception of the time period dummy which, which was borderline significant (OR 1.30; 95% CI 0.95–1.78; *p*-value 0.096). The coefficient estimates also remained robust in Model 3; and the estimated coefficient for the marital status dummy indicated that single individuals were more likely to be in receipt of a DP (OR 1.80; 95% CI 1.19–2.72; *p*-value 0.005) than their married counterparts and those living in a registered partnership.

**Table 5:** Logistic regression models with DP as outcome, 18–39-year-olds, N = 5306 (weighted estimates)

	Model 1			Model 2			Model 3		
	OR	95% CI	*p*-value	OR	95% CI	*p*-value	OR	95% CI	*p*-value
Sex									
Female	1			1			1		
Male	1.01	0.76–1.34	0.958	1.03	0.75–1.41	0.849	1.01	0.76–1.34	0.958
Working partner in household									
Yes	1			1					
No	2.11	1.51–2.94	0.000	2.09	1.49–2.92	0.000			
Child aged 0–14 years in household									
Yes	1			1			1		
No	2.15	1.48–3.12	0.000	2.21	1.53–3.21	0.000	1.97	1.31–2.96	0.001
Country of birth									
Outside of Switzerland	1			1			1		
Switzerland	2.68	1.87–3.84	0.000	2.60	1.82–3.73	0.000	2.51	1.75–3.60	0.000
Age	1.16	1.12–1.19	0.000	1.16	1.13–1.19	0.000	1.15	1.12–1.19	0.000
Highest degree attained									
Upper secondary or tertiary	1						1		
Lower secondary	3.26	2.24–4.76	0.000				3.42	2.34–5.00	0.000
Income during 4 years prior to interview									
Yes	1			1			1		
No	3.94	2.70–5.75	0.000	3.80	2.57–5.62	0.000	4.14	2.84–6.04	0.000
Chronic illness									
No	1			1			1		
Yes	4.52	2.83–7.19	0.000	4.33	2.71–6.94	0.000	4.52	2.84–7.19	0.000
Long-term activity limitation									
Limited but not severely	1			1			1		
Severely limited	4.24	3.10–5.81	0.000	4.32	3.14–5.93	0.000	4.21	3.08–5.76	0.000
Time period									
2012–2015	1			1			1		
2010–2011	1.42	1.05–1.92	0.022	1.30	0.95–1.78	0.096	1.43	1.05–1.93	0.021
Learnt occupation									
Health, education, culture, and science				1					
Not applicable				5.93	3.72–9.46	0.000			
Agriculture				2.61	0.95–7.17	0.063			
Manufacturing				3.59	1.91–6.71	0.000			
Technical activities and ICT				0.71	0.28–1.83	0.482			
Construction and mining				1.08	0.40–2.93	0.873			
Trade and transport				2.14	1.16–3.96	0.015			
Personal services				2.26	1.00–5.09	0.050			
Business and administration				2.44	1.40–4.23	0.002			
Not classifiable				1.46	0.62–3.41	0.386			
Marital status									
Married or in registered partnership							1		
Single							1.80	1.19–2.72	0.005

Source: SESAM, FSO

Table 6 reports the results of the regression analysis for the subsample aged 25–39. The same pattern emerged as in the 18–29-year-old sample, with the exception of the time period indicator and ‘Agriculture’. The coefficient estimates on the latter two variables remained robust in magnitude but were statistically not significant.

**Table 6:** Logistic regression models with DP as outcome, 25–39-year-olds, N = 4170 (weighted estimates)

	Model 1			Model 2			Model 3		
	OR	95% CI	*p*-value	OR	95% CI	*p*-value	OR	95% CI	*p*-value
Sex									
Female	1			1			1		
Male	0.92	0.67–1.27	0.616	0.91	0.64–1.30	0.611	1.03	0.75–1.41	0.866
Working partner in household									
Yes	1			1					
No	2.08	1.47–2.93	0.000	2.07	1.46–2.92	0.000			
Child aged 0–14 years in household									
Yes	1			1			1		
No	2.15	1.45–3.17	0.000	2.17	1.48–3.18	0.000	2.00	1.30–3.06	0.002
Country of birth									
Outside of Switzerland	1			1			1		
Switzerland	3.66	2.42–5.55	0.000	3.44	2.29–5.16	0.000	3.44	2.27–5.22	0.000
Age	1.09	1.04–1.14	0.000	1.09	1.04–1.13	0.000	1.09	1.04–113	0.000
Highest degree attained									
Upper secondary or tertiary	1						1		
Lower secondary	3.99	2.58–6.16	0.000				4.17	2.69–6.47	0.000
Income during 4 years prior to interview									
Yes	1			1			1		
No	5.98	4.28–8.35	0.000	5.78	4.08–8.19	0.000	6.24	4.46–8.73	0.000
Chronic illness									
No	1			1			1		
Yes	4.52	2.68–7.62	0.000	4.17	2.47–7.05	0.000	4.50	2.68–7.57	0.000
Long-term activity limitation									
Limited but not severely	1			1			1		
Severely limited	3.35	2.35–4.78	0.000	3.32	2.33–4.72	0.000	3.38	2.37–4.80	0.000
Time period									
2012–2015	1			1			1		
2010–2011	1.27	0.91–1.78	0.154	1.19	0.84–1.68	0.330	1.29	0.92–1.80	0.141
Learnt occupation									
Health, education, culture, and science				1					
Not applicable				6.57	3.99–10.80	0.000			
Agriculture				2.07	0.70–6.09	0.188			
Manufacturing				3.27	1.74–6.16	0.000			
Technical activities and ICT				0.76	0.29–1.96	0.568			
Construction and mining				1.44	0.50–4.13	0.496			
Trade and transport				2.18	1.16–4.08	0.015			
Personal services				2.73	1.21–6.15	0.016			
Business and administration				2.60	1.46–4.64	0.001			
Not classifiable				1.51	0.62–3.69	0.369			
Marital status									
Married or in registered partnership							1		
Single							1.73	1.13–2.64	0.011

Source: SESAM, FSO

The estimation results for the subsample excluding partial DP recipients, presented in Table 7, are also in line with those in our benchmark sample, with two exceptions in Model 2. First, the coefficient estimate on the time period indicator was similar in magnitude and was statistically significant (OR 1.49; 95% CI 1.01–2.20; p-value 0.042). Second, the coefficient estimate on ‘Trade and transport’ remained robust in magnitude but was not statistically significant (OR 1.82; 95% CI 0.80–4.12; *p*-value 0.152).

**Table 7:** Logistic regression models with full DP as outcome, 18–39-year-olds, *N* = 5196 (weighted estimates)

	Model 1			Model 2			Model 3		
	OR	95% CI	*p*-value	OR	95% CI	*p*-value	OR	95% CI	*p*-value
Sex									
Female	1			1			1		
Male	1.09	0.76–1.54	0.646	1.17	0.80–1.71	0.429	1.23	0.86–1.75	0.256
Working partner in household									
Yes	1			1					
No	3.12	1.95–5.00	0.000	3.24	2.02–5.21	0.000			
Child aged 0–14 years in household									
Yes	1			1			1		
No	2.74	1.62–4.62	0.000	2.80	1.68–4.64	0.000	2.22	1.29–3.85	0.004
Country of birth									
Outside of Switzerland	1			1			1		
Switzerland	2.95	1.90–4.59	0.000	2.87	1.84–4.48	0.000	2.63	1.70–4.06	0.000
Age	1.15	1.11–1.20	0.000	1.16	1.12–1.20	0.000	1.15	1.11–1.19	0.000
Highest degree attained									
Upper secondary or tertiary	1						1		
Lower secondary	4.07	2.55–6.49	0.000				4.36	2.72–6.99	0.000
Income during 4 years prior to interview									
Yes	1			1			1		
No	4.38	2.65–7.23	0.000	4.13	2.41–7.05	0.000	4.73	2.87–7.82	0.000
Chronic illness									
No	1			1			1		
Yes	3.59	2.08–6.20	0.000	3.47	1.99–6.05	0.000	3.61	2.10–6.21	0.000
Long-term activity limitation									
Limited but not severely	1			1			1		
Severely limited	4.89	3.32–7.19	0.000	5.16	3.50–7.60	0.000	4.79	3.26–7.04	0.000
Time period									
2012–2015	1			1			1		
2010–2011	1.68	1.15–2.44	0.007	1.49	1.01–2.20	0.042	1.67	1.15–2.43	0.008
Learnt occupation									
Health, education, culture, and science				1					
Not applicable				9.07	4.68–17.57	0.000			
Agriculture				3.24	0.90–11.67	0.072			
Manufacturing				3.04	1.30–7.10	0.010			
Technical activities and ICT				0.89	0.25–3.15	0.854			
Construction and mining				0.96	0.25–3.73	0.949			
Trade and transport				1.82	0.80–4.12	0.152			
Personal services				3.33	1.17–9.50	0.025			
Business and administration				3.18	1.50–6.74	0.002			
Not classifiable				1.39	0.43–4.57	0.583			
Marital status									
Married or in registered partnership							1		
Single							2.68	1.58–4.53	0.000

Source: SESAM, FSO

## Discussion

### Main findings

This study explored the associations between demographic, socioeconomic, and health factors and DP in young adults with long-term activity limitation living in Switzerland using data from the 2010–2015 SESAM cross-sections; a unique dataset linking microdata from the SLFS and different social insurance registers. Our estimates revealed that young adults living without a working partner and without a child aged 0–14, born in Switzerland, of higher age, having completed at most lower secondary school, lacking income throughout the four-year period prior to interview, and reporting chronic illness and severe long-term activity limitation had higher odds of DP. Our findings were robust to alternative specifications and subsamples; and the results of the alternative specifications showed that marital status and learnt occupation were statistically significantly associated with DP.

Whereas studies examining factors associated with DP in the general population abound, there are fewer studies focusing on young adults; despite the fact that young adults represent a unique and special at-risk group. We will thus discuss our results in the context of the scarce existing Swiss and international evidence on young adults; and also draw comparisons to the literature on middle-aged individuals and the general population. The comparisons cover other OECD countries, focusing on European countries with a GDP per head of population similar to Switzerland’s [40]. These European countries are also comparable to Switzerland in that the employment rate of individuals with disability have been higher than the OECD-average in the late 2000s [3]. Nevertheless, the cross-country comparisons should be interpreted with caution in light of differences in institutional settings.

First of all, we draw on the results of a recent Swiss report [41] examining the risk factors of DP in young adults aged 18–29, with a mental illness diagnosis, and living in Switzerland. Note that the findings are not entirely suitable for a comparison to our study not only given the less inclusive sample in terms of diagnosis, but also the different set of socioeconomic and health-related variables included in the analysis. Data limitations arising from the small sample size (*N* = 500) and the data collection procedure further aggravate comparison. Nevertheless, similarly to the present study, low educational attainment was found to be a statistically significant factor for DP in the Swiss report, and no statistically significant association was found between sex and DP. In fact, low educational attainment has been described as a risk factor for DP, independent of the cohort under analysis [10, 20, 22]. For example, a Norwegian and a Swedish study demonstrated the relationship between low educational attainment and DP in individuals aged 18 to 66 and in individuals aged 17 to 65, respectively [10, 22]. When stratifying the sample by age, the latter study revealed that low education was more strongly associated with the granting of a DP in young adults aged 17 to 45 than in individuals aged 46 to 65 [10].

Our results concerning learnt occupation, an alternative measure of education, shed more light on the association between education and DP. First, our results indicating that those without an occupational qualification have the highest odds of DP confirm our findings on broad educational levels. Second, the relatively high odds of DP for occupational qualifications within the ‘Business and administration’ category may seem counterintuitive given the inverse association between educational level and DP and the high share of tertiary graduates within ‘Business and administration’. Occupational and workplace factors may explain this finding, assuming that the respective individuals have worked long enough in their learnt occupation for the latter factors to play a role. In particular, there is evidence in middle-aged Swedish workers and Finnish male workers for the positive association between mentally strenuous work, time pressure, neck and back strain and DP [20, 42]; characteristics which are likely to be dominant in the banking, insurance and legal sectors as well as in managerial positions. Physical and repetitive strain, low decision latitude, and noise exposure may in turn explain the high odds of DP in ‘Manufacturing’, ‘Trade and transport’, and ‘Personal services’ occupations.

Marriage/living in a partnership is supposed to protect against marginalization [10, 17, 21, 43], and being married has been documented to be positively related to mental and physical well-being [44]. Our results for young adults augment these findings: independent of whether partnership is defined by marital status or by the presence of a working partner in the household, a protective effect was found.

The finding that individuals living without a child aged 0–14 are more likely to receive a DP is consistent with a Swedish study analysing men and women aged 18–59 [17] as well as with the results of another Swedish study according to which single people aged 45 and younger without children have higher odds of DP than their married/cohabiting counterparts with children [10]. The factor ‘child’ can be seen from two perspectives. First, the presence of children in the household may be interpreted as a protective effect. As such, our findings augment those indicating children’s protective effect with regard to suicidal behavior [9] as well as the general finding that women with family or caring commitments generally have less adverse health effects, possibly because they have better alternative social roles [45]. An additional explanation in the case of mothers may lie in institutional factors; that is, the so-called combined method employed by the FSIO for the time period under analysis. The combined method was applied in cases of part-time employment to calculate the degree of disability assessing the reduction in the capacity to engage in paid employment and to perform household tasks and childcare, separately [46]. The combined method allegedly discriminated against part-time employees as it generally led to a lower degree of disability for part-time employees than for full-time employees [46]; affecting especially mothers with reduced working hours, to whom the combined method was overwhelmingly applied [47, 48]. In fact, in the one Swiss case (di Trizio v. Switzerland) lodged with the European Court of Human Rights (ECtHR) in 2009 ‘the applicant complained mainly of the fact that the ‘combined method’ applied in order to calculate her degree of disability had resulted in her being refused a benefit because she had worked part-time’ [48]. The ECtHR came to the conclusion that combined method as applied in the di Trizio v. Switzerland case indeed suggested the presence of ‘indirect discrimination’ [48]. In Switzerland the vast majority of women (and a growing number of men) work part-time after the birth of their child [49]. As such, our finding appears to be in line with the disadvantaging effect resulting from the application of the combined method [47, 48]. However, further research is needed to study this phenomenon; especially in light of the recent replacement of the combined method by a new calculation method for part-time employees [44, 45].

Higher age, an indicator of health status, has been widely documented to be a risk factor for DP in the working-age population [17, 20, 43, 50]. Given the relatively narrow age range in our sample(s), it is not surprising that age was associated with just slightly higher odds of DP. Our finding is in agreement with and most comparable to two Swedish studies which found that (1) individuals aged 30–39 had a higher risk of DP than those aged 16–29 [17] and that (2) individuals aged 26–35 and 36–45 had a higher risk of DP than those aged 17–25 [10].

That young adults born in Switzerland were more likely to receive a DP than those born outside of Switzerland may seem surprising in light of the international literature implying the opposite in the general population in Norway [10], Sweden [17], Germany [21], and the UK [21]; in middle-aged Swedish workers [42]; and even in a relatively small and specific sample of young adults initially sick-listed with back diagnosis living in Sweden [51]. In our estimates, the likelihood of DP application and DP receipt cannot be disentangled. It is thus possible that young adults born in Switzerland are more likely to apply for a DP – potentially because they are more familiar with the DP system – and just as likely to receive a DP as their counterparts born abroad. Accordingly, after controlling for selection into DP application, the association between DP and country of birth may change. Furthermore, the familiarity with the DP system and thus the likelihood of applying for a DP could be increasing in the time spent in Switzerland for individuals born outside of Switzerland. This in turn may lead to different associations between country of birth and DP in younger and older cohorts. In fact, no association was found between country of birth and DP receipt in individuals aged 15 to 64 living in Switzerland [21].

Although we cannot disentangle the various types of income which make up our binary income measure, we can conclude that those with at least one recorded income had been working or seeking employment at least once within a four-year period. Subsequently, our results augment the evidence on the positive association between unemployment and DP in the general population in Sweden [17, 43] and in Germany [21]; and in middle-aged Finnish male workers [20]. Long-term sickness absence spells have been shown to be associated with DP in Finland [52] and in Sweden [6, 53]; and there is evidence that the replacement rate of mandatory sickness absence insurance is a key determinant of duration of sickness absence spells [54]. Subsequently, within young adults with long-term activity limitations, those with past employment experience but long-term sickness absence spells may be at risk of future DP.

That chronic morbidity was positively associated with DP in young adults was expected in the light of a recent EU report on the employment opportunities of individuals with chronic illness [55]. According to this report, people with chronic disease have a high risk of unemployment and inactivity in the EU; with the largest proportion of people typically outside the labour market such as in receipt of DP. While the transition from employment to inactivity/unemployment has been documented to be quick for those with chronic illness, the transition from inactivity/unemployment to employment seems to be particularly complicated for those affected by chronic illness. The EU report outlines numerous factors at the employer-level which account for this uneven employment transition path. For instance, employers in Sweden are concerned about the legal obligations to facilitate the return to work including workplace adaptation requirements. Moreover, evidence from a number of EU countries illustrates that workers with chronic diseases are particularly affected by discrimination and prejudice at work. In addition, not everybody who needs to be supported is actually supported, despite the fact that national legislation often gives the right to a reasonable adaptation of the workplace contents. To what extent these workplace factors apply to the Swiss context should be a focus of further research in order to understand how to enhance the employment opportunities of the particularly vulnerable group of young adults with chronic illness, potentially differentiating between those with mental and physical illness.

Our second health measure, long-term activity limitation, captured an additional health domain independently of the type of activity, the specific life situation, and the kind of health problem [31]. Impediments in daily activities have been documented to be associated with DP receipt in those aged 15–64 living in Switzerland and the UK [21]. Our results augment these findings in identifying the *severity* of the long-term activity limitation as a factor for DP in young adults.

### Methodological considerations

A strength of or study is the large sample of young adults enabling us to shed light on a particularly at-risk group rather than presenting aggregated results for the general population. Moreover, the extensive SESAM data allowed us to control for a wide range of demographic and socioeconomic factors. Furthermore, a homogeneous group of non-DP recipients in terms of employment status could be selected in the current study; an aspect deemed essential but not feasible due to data limitations in the one recent Swiss report focusing on the same research question [41].

The dataset does have its limitations, however. First, sufficiently detailed information concerning the individual’s health was not available in our dataset. In particular, we could not control for the individual’s medical diagnosis; a factor which has been documented to be associated with labour market outcomes [56] and DP [17, 20, 41]. The second limitation concerns the lack of information on DP application; we do not know whether the individuals in our sample have applied for DP or not and whether they are in the process of DP application. Consequently, DP receipt and DP application cannot be disentangled in the current study. While the sample size was large compared to the Swiss report mentioned above [41], it was not large enough to employ more disaggregated categories for highest educational attainment. The Norwegian study [22] for instance was able to show a reduced risk for DP for those with a PhD education relative to those with merely a university education. Finally, given the small number of individuals in numerous learnt occupational categories and the resultant effect on the statistical significance of our results, a larger sample would be valuable in order to re-examine the association between learnt occupations and DP.

Overcoming the limitations described above is subsequently the focus of our future research; preferably by relying on data which extends to more recent SESAM cross-sections and links information on DP application, DP history, and medical diagnosis.

## Conclusions

In terms of socioeconomic characteristics, the most vulnerable group included young adults who (i) completed at most lower secondary school and (ii) were without income for a relatively long period of four years. These results imply that early intervention should focus on (i) the attainment of vocational and academic qualifications beyond the lower secondary level and (ii) facilitating labour market integration. Avoiding school-dropout as well as supporting students with long-term activity limitations to complete upper secondary education falls in the responsibility of educational and health care systems. Our results concerning learnt occupation and health factors stress the importance of both early and ongoing vocational and career counselling in order to achieve an optimal match between individual vulnerability and occupational and workplace characteristics.

## Data Availability

The data that support the findings of this study are available from the Swiss Federal Statistical Office but restrictions apply to the availability of these data, which were used under license for the current study, and so are not publicly available.

## Abbreviations

95% CI: 95% confidence interval
CCO: Swiss Central Compensation Office
DP: Disability pension
ECtHR: European Court of Human Rights
FSIO: Swiss Federal Social Insurance Office
FSO: Swiss Federal Statistical Office
MEHM: Minimum European Health Module
NCCR LIVES: Swiss National Centre of Competence in Research LIVES – Overcoming vulnerability: Life course perspectives
OR: Odds ratio
SECO: State secretariat for economic affairs
SESAM: Social protection and labour market
SHAPE: Swiss System for Household and Personal Statistics
SLFS: Swiss Labour Force Survey
SSCO: Swiss Standard Classification of Occupations
